# The use of surveillance and preventative measures for methicillin-resistant *staphylococcus aureus* infections in surgical patients

**DOI:** 10.1186/2047-2994-3-18

**Published:** 2014-05-14

**Authors:** Kevin T Kavanagh, Lindsay E Calderon, Daniel M Saman, Said K Abusalem

**Affiliations:** 1 Health Watch USA, 3396 Woodhaven Dr, Somerset, KY 42503, USA; 2 Eastern Kentucky University, Richmond, KY, USA; 3 Essentia Institute of Rural Health, Duluth, MN, USA; 4 University of Louisville, Louisville, KY, USA

## Abstract

The Agency for Healthcare Research & Quality (AHRQ) found that Methicillin-resistant *Staphylococcus aureus* (MRSA) is associated with up to 375,000 infections and 23,000 deaths in the United States. It is a major cause of surgical site infections, with a higher mortality and longer duration of care than Methicillin-sensitive *Staphylococcus aureus*. A multifactorial bundled approach is needed to control this epidemic, with single interventions unlikely to have a significant impact on attenuating MRSA infection rates.

Active surveillance has been studied in a wide range of surgical patients, including surgical intensive care and non-intensive care units; cardiac, vascular, orthopedic, obstetric, head and neck cancer and gastrostomy patients. There is sufficient evidence demonstrating a beneficial effect of surveillance and eradication prior to surgery to recommend its use on an expanded basis.

Studies on MRSA surveillance in surgical patients that were published over the last 10 years were reviewed. In at least five of these studies, the MRSA colonization status of patients was reported to be a factor in preoperative antibiotic selection, with the modification of treatment regiments including the switching to vancomycin or teicoplanin in MRSA positive preoperative patients. Several authors also used decolonization protocols on all preoperative patients but used surveillance to determine the duration of the decolonization.

Universal decolonization of all patients, regardless of MRSA status has been advocated as an alternative prevention protocol in which surveillance is not utilized. Concern exists regarding antimicrobial stewardship. The daily and universal use of intranasal antibiotics and/or antiseptic washes may encourage the promotion of bacterial resistance and provide a competitive advantage to other more lethal organisms.

Decolonization protocols which indiscriminately neutralize all bacteria may not be the best approach. If a patient's microbiome is markedly challenged with antimicrobials, rebuilding it with replacement commensal bacteria may become a future therapy.

Preoperative MRSA surveillance allows the selection of appropriate prophylactic antibiotics, the use of extended decolonization protocols in positive patients, and provides needed data for epidemiological studies.

## Review

### The impact of MRSA

Despite over a decade of interventions, Methicillin-resistant *Staphylococcus aureus* (MRSA) remains a significant and all too common pathogen encountered in the U.S. Healthcare system. Extrapolating data from selected counties from nine states, the Centers for Disease Control and Prevention (CDC) estimated that in the United States MRSA caused over 80,000 “invasive” infections and over 11,000 deaths in 2011 [1]. This data represents documented severe infections, requiring a positive MRSA culture from a normally sterile site, such as blood cultures associated with bacteremia. Using billing data from U.S. hospitals from 2011, the Agency for Healthcare Research & Quality (AHRQ) found that more than 460,000 hospitalizations had a MRSA diagnosis, with 23,000 deaths associated with these admissions [2]. MRSA is a leading cause of healthcare associated infections [1,3], and patients undergoing a surgical procedure are at an increased risk of acquiring these infections [1]. Kaye et al. reported that the average immediate economic impact from treating a deep incisional or organ space surgical site infection is $43,970 in patients 65 years and older [3], not counting possible long term treatment and disability. Unlike other adverse events, eliminating the occurrence of infections cannot be completely controlled and regulated by a single facility, as infection control measures at one facility may well affect and contribute to the MRSA prevalence in other nearby facilities [4]. Many different types of facilities are at risk, along with their service communities, making the need to control healthcare associated infections even more imperative. Colonization rates of *Staphylococcus aureus* (*S. aureus*) in the United States are approximately 30% [5]. The rate of MRSA colonization in patients admitted to hospitals tend to range from 1.3% to 7.6% [6-8], with colonized patients displaying a higher risk of developing a MRSA infection [9-13].

Control of this epidemic is multifactorial, with single interventions unlikely to have a significant impact on attenuating MRSA infection rates. Hand hygiene is of importance, but institutions that only focus on this intervention are unlikely to be successful in controlling MRSA infections. Surgeons performing even simple office procedures using sterile gloves alone know the difficulties in maintaining a sterile field. As observed by Lee et al. [14], a bundle approach was required and neither active surveillance or hand hygiene alone produced significant reductions in MRSA infections in surgical wards. Using a screening and decolonization protocol, Rao et al. observed that none of the 147 (methicillin-sensitive *Staphylococcus aureus*) MSSA and 17 MRSA colonized patients developed an infection post joint arthroplasty [15].

Active surveillance for MRSA has been studied on a wide range of surgical patients, including surgical intensive care and non-intensive care units; cardiac, vascular, orthopedic, obstetric, head and neck cancer and gastrostomy patients [14,16-18]. The vast amount of the research has centered on the inpatient setting, of which a combinational or bundled approach has emerged as a superior intervention in preventing the spread of MRSA infections [14,18].

### Preventative measures for MRSA infections in surgical patients

Surgeons encounter two different scenarios where MRSA preventative measures are needed. The first is for surgery patients admitted to surgical units or intensive care units where universal surveillance testing and isolation vs universal daily decolonization have been advocated. The second is for patients admitted for same-day surgery where preoperative MRSA screening and decolonization can by performed on an outpatient basis, eliminating the need for isolation.

The prevention of the spread of MRSA in surgical units is of critical importance. Prolonged contact with the healthcare setting increases the possibility of patients contracting MRSA from bacteria brought into the facility by other patients. Thus, the longer the contact and the sicker the patient the greater the concern.

One of the factors that may have impeded the adoption of active surveillance protocols [19] was the highly quoted study published by Harbarth et al. [20]. This study was referred to in a U.S. Congressional hearing as one of the reasons why the effects of screening are poorly understood [21,22] and by The Society for Healthcare Epidemiology of America/Healthcare Infection Control Practices Advisory Committee (SHEA/HIPAC) as a reason why recommendations regarding active surveillance testing cannot be made [23].

The Harbarth et al., study was well-controlled and produced negative findings in regard to the utilization of active surveillance methods. However, it had methodological limitations [19] and appeared to have identifiably known deficiencies in implementation: 31% of the patients were identified as MRSA carriers after, not before, the surgery, and only 43% of patients who were known to be carriers before surgery received preoperative antibiotics effective against MRSA [20]. Of the patients who developed a MRSA infection and were known carriers prior to surgery, only 66% received prophylaxis against MRSA and almost 60% did not receive optimal decolonization of MRSA prior to surgery [20]. In addition, the study was contradicted by a well-controlled, large multi-national, multi-institutional study published by Lee et al. [14], (whose corresponding author was Harbarth) which reported surveillance along with hand hygiene and targeted eradication of MRSA to be successful in preventing MRSA surgical infections.

There are at least 19 studies over the last decade that observed a beneficial effect of active surveillance in the prevention of MRSA infections [14-16]. Seventeen of these studies were reviewed in a recent AHRQ publication [16]. We reviewed these 19 studies and found that in 14, observed benefits of active surveillance reached statistical significance. However, with the exception of Lee et al. [14], the studies that demonstrated a statistically significant effect on MRSA have been criticized for deficiencies in design, not controlling confounding variables and/or secular trends [16,19]. In other words, the majority of the studies were a before and after design and there is a possibility that an unknown factor may have been present or introduced as these studies were conducted. Confounding factors may vary results in either direction. For example, the implementation of another seemingly unrelated protocol might produce an unexpected augmentation of the intervention’s positive results or the increase in the rates of MRSA in the community might produce an unexpected mitigation of the observed results. However, dominant impacting factors causing positive results across so many studies is unlikely.

A review of the literature regarding MRSA active surveillance in surgical patients was published by AHRQ in 2013 and failed to make recommendations, despite multiple studies observing that active surveillance reduces MRSA infections [16]. Taken together, the negative study published by Harbarth et al. [20], and the lack of control for unknown possible confounding factors in the remaining studies, appeared to be reasons that prevented AHRQ from making recommendations.

If the studies with known unadjusted confounding factors (the factor that biased the results is evident) are disregarded and the myriad of positive studies with a possible unknown confounding factor are then considered, the case for the use of active surveillance would be strengthened.

This is not to say that as new treatments emerge protocols should not be changed, but not implementing or having the best practice protocols in place during a rampant epidemic may be a leading cause for the inability to control multi-resistant organisms in the United States. This problem was outlined in a 2008 report from the Government Accountability Office (GAO), which found a need for the U.S. Department of Health and Human Services to prioritize the almost 1200 recommended prevention practices, of which over 500 are strongly recommended [24]. Recently, Drees et al. [25] found highly variable definitions and management protocols for Multidrug resistant gram-negative bacilli and concluded that this variation might contribute to the emergence of these bacteria.

Different prevention protocols are indicated for patients admitted the day of surgery. In this setting, emphasis can be placed on eradication of the carrier state before surgery. A common protocol was the use of intranasal mupirocin and a daily bathing with either chlorhexidine [14,15,26,27] or triclosan [6,28,29] for 5 days. Several studies required three consecutive negative swabs before MRSA eradication was confirmed and the patient could proceed for elective surgery [6,30]. Many of these patients would not have to be isolated, but one could argue that they were effectively isolated from the treatment facility during outpatient decolonization.

There is ample evidence for the beneficial effect of active surveillance and eradication prior to surgery. Why not then subject all patients to the decolonization/eradication protocols and not perform surveillance? There are three concerns with this approach. The first is regarding the selection of antibiotics for preoperative prophylaxis, the second is the promotion of bacterial resistance on the targeted bacteria, and the third is the effect on the microbiome of both the patient and facility.

### Selection of antibiotics for preoperative prophylaxis

All studies on surgical patients analyzed by AHRQ and those published by Lee et al. [14], and Rao et al. [15], were reviewed. At least five of these studies varied the type of preoperative antibiotic depending upon MRSA surveillance results [14,15,27,29,31]. The antibiotic was either ‘modified’ [14], switched to vancomycin [15,27,31] or combined with teicoplanin [29]. This is important, because as observed by Gupta et al. [11], vancomycin is associated with an increase in surgical site infections in non-MRSA carriers but not in MRSA carriers. An extensive review of this subject was conducted by Crawford et al. [32], who observed that “…the suspected weaker activity against MSSA” has been one of the factors in preventing the routine use of vancomycin for preoperative prophylaxis.

Even though MRSA has been observed to have a higher mortality and a longer length of treatment than MSSA [32], MSSA is an important pathogen and has been observed to be an important causative organism in hip and knee arthroplasty [15]. If MSSA is detected preoperatively, eradication prior to elective surgery has been observed to reduce surgical site infections [33].

Several authors also used decolonization protocols on all preoperative patients, but used surveillance to determine the duration of the decolonization. For example: In MRSA carriers, Jog et al. [29], used surveillance to extend the decolonization period with mupirocin and triclosan to five days, (United Kingdom’s National Health Services recommends five days of decolonization for newly identified carriers [34]), and to substitute preoperative antibiotics gentamicin and teicoplanin for gentamicin and flucloxacillin. The authors observed a significant reduction in MRSA infections.

However, these protocols also relate to the second concern, the promotion of bacterial resistance, which may take years or decades to emerge [35]. Of increased concern is the eradication protocols that are performed on all patients every day, regardless of colonization status, in an inpatient setting or unit. Many decolonization protocols use both an intranasal antibiotic and an antiseptic body wash. This results in a repeated and prolonged exposure of both the patient’s and facility’s microbiome to the antimicrobial agents.

### Emergence of resistance in the targeted bacteria

#### ***Use of intranasal antibiotics***

Concern exists regarding the frequent use of intranasal antibiotics in decolonization protocols. Jog et al. [29], and Walsh et al. [31], warn of the possible development of mupirocin resistance. In some facilities, such as nursing homes, mupirocin resistance can approach 31% in MRSA carriers [36]. There are also doubts regarding the effectiveness of decolonization without the use of topical intranasal antibiotics and the vast majority of studies use intranasal mupirocin in their decolonization protocols. However, Derde et al. [37] observed decrease MRSA infection with a protocol that used chlorhexidine bathing that did not incorporate intranasal mupirocin.

#### ***The use of antiseptic washes***

The use of antiseptic washes are commonly prescribed preoperatively. In this sense, it is the beginning of prepping a site before surgery. When washing is used for decolonization it involves the entire body, as the classical surgical prep is often confined to the surgical site. The concept of antimicrobial stewardship is important in the discussion of this topic. No one advocates not prepping patients for surgery, and use of an antiseptic wash preoperatively at home is not likely to affect the microbiome of a facility miles away. Controversy exists as to whether body washes are used for one day pre-operatively, five days pre-operatively or if they should be used on a daily basis on everyone who is admitted to a surgical intensive care unit. In addition, there is also controversy on whether or not to use an antiseptic or just a detergent [38].

Two recent prominent studies have evaluated the daily and universal use of chlorhexidine decolonization in preventing infections in ICUs. The first, by Haung et al. [39], using both chlorhexidine and intranasal mupirocin, did not achieve a statistically significant reduction for MRSA bloodstream infections, but did for the reduction in MRSA isolates and for reduction of bloodstream infections in the “Any pathogen” category. However, in the “Any Pathogen” category, by far the greatest reduction observed was for commensal bacteria. The clinical trial originally was to also measure central line-associated bloodstream infections (CLABSI) and MRSA urinary cultures, but CLABSI data was dropped due to inability to acquire standardized denominators and data on the revised metric, urinary infections, is still pending [40]. A second article by Derde et al. [37], found that MRSA acquisition decreased with improved hand hygiene and unit-wide decolonization of patients with chlorhexidine. However, MRSA chlorhexidine resistance-associated genes was observed in 13% to 14% and over the short period of the study, resistance had a statistically non-significant increase of 11.2% (14 of 110 verses 16 of 113) [37]. In addition, there was not a reduction in acquisition of highly resistant *Enterobacteriaceae* or vancomycin-resistant *enterococci*.

Failure of decolonization therapy in MRSA carriers has been observed by Lee et al. [41] who found a significant increase in persistent MRSA carriage in patients with a combined low-level mupirocin and genotypic chlorhexidine resistance. Although the exact mechanism is not entirely understood, no matter how strong a topical antiseptic is, a no kill zone may be present. This includes areas in both the facility and in hard to reach areas of the patient. In addition, if there is only a bacterial static effect, reemergence could then occur once the concentration of the antiseptic falls.

#### ***The use of preoperative antibiotics***

Similar concerns regarding the development of resistance exist with the parenteral administration of prophylactic antibiotics. The development of gram positive resistance to vancomycin along with possible weaker effectiveness against MSSA have been major reasons not to routinely use prophylaxis [32].

#### ***Effects on the microbiome***

We live in harmony with commensal bacteria and are harmed by pathogenic bacteria. The importance of the types of bacteria residing on and in the body cannot be overstated. For example, in surgical patients Gupta et al. [11], observed that post-operative infection rates may vary after the use of vancomycin prophylaxis depending upon the colonization status of the patient.

The effects of antimicrobial agents on the microbiome is not only that bacteria may develop antimicrobial resistance, but also that a selective advantage may be produced for other bacteria, sometimes even more virulent, that then take the place of the eradicated pathogen. For example, the microbiome of the gut is attenuated and challenged with the administration of antibiotics. Post-antibiotic diarrhea may develop and be a sign of a *C. difficle* infection. It has been reported that *C. difficile* infections can be reduced by over 70% by restricting the use of ceftriaxone and ciprofloxacin [42] and, thus, helping to preserve the microbiome of the patients.

A building’s microbiome is a highly complex and dynamic system [43]. As discussed by Kelley and Gilbert [43] the climate, materials, and building design could have unexpected and interesting consequences for the selection and growth of microbes. When the microbiome of a facility enriches certain traits, “unnatural selection” occurs and a few bacterial groups will become dominant. Such selection has been observed to occur in facilities ranging from hospitals [43] to cheese factories [44].

Many studies reporting rates of resistance to topical antimicrobials only evaluate the organism the study targets for reduction. But, a very real theoretical concern is that the overuse of antibiotics and antiseptics may well select for other resistant bacterial strains and change the pathological flora in the building’s environment. For example, bacterial spores are resistant to chlorhexidine body washes [45] which may give a selective advantage to *C. difficile*. In addition, reduced susceptibility to chlorhexidine has been observed in Carbapenem-resistant *Enterobacteriaceae* and has been postulated by one author to promote the extremely-drug-resistant epidemic strain of *Klebsiella pneumonia*[46].

Indiscriminately neutralizing a patient’s bacteria may not be the best approach. If a microbiome is markedly challenged with antimicrobials, rebuilding it with replacement commensal bacteria may become a future therapy. This technique is already taking hold with the resurgence of research into probiotics and fecal transplants to prevent *C. difficile* infections [47].

Currently, surveillance is limited to targeted pathologic bacteria that have high endemic levels in the community and/or in patients at higher risk for carrying them. However, someday it may become important and feasible to know the entire microbiome of patients. This may even become an essential part of a routine history and physical, and the surveying for chronic infections or colonizations by pathologic bacteria may become of great importance. If our microbiome becomes out of balance unexpected diseases such as gastric ulcers by *Helicobacter pylori*, chronic plaque psoriasis from recurrent streptococcal infections [48], and rheumatoid arthritis from Porphyromonas *gingivalis* may emerge [49].

## Conclusion

The vast majority of studies appear to support that surveillance followed by targeted intervention is effective in reducing surgical site infections. Strong consideration should be given to classifying surveillance for MRSA as a U.S. Preventive Services Task Force (USPSTF) Grade B recommendation, in that “there is a high certainty that the net benefit is moderate and there is a moderate certainty that the net benefit is moderate to substantial” [50]. Indications for active surveillance testing need to be changed from using on high-risk populations to performing on all except low-risk populations.

The United Kingdom’s National Health Service’s best practices include MRSA screening of orthopaedics, cardiothoracic and neurosurgical patients; emergency orthopaedic and trauma admissions; critical care admissions; all elective surgical patients; previous MRSA carriers; oncology/chemotherapy patients; renal patients and patients admitted from high risks settings such as nursing and care home and all emergency admissions [34]. And “the logical conclusion of risk factor assessments and the results of modelling studies is that the most appropriate approach to the reduction in MRSA carriage in the population, and resultant MRSA infections, is the universal screening of all admissions to hospital (either at pre-admission clinics for elective admissions or immediately on admission for emergency admissions)” [34].

In the future, knowing the patient’s microbiome may become part of a standard physical examination However, for now, respecting the microbiome and determining carriers of common pathogens of patients treated at a facility is a prudent practice. Surgeons should strongly consider decolonization of patients before a surgical procedure and active surveillance testing for common pathogens treated at their institution so the principles of antibiotic stewardship can be followed and proper antibiotics given.

Universal decolonization versus surveillance and selective decolonization is an area in need of more research. All surgical patients undergo a surgical scrub and draping, effectively a localized decolonization, before a surgical incision is performed. And although universal decolonization at home will not be expected to affect a facilities’ micorbiome, exposing hospital rooms to decades of daily cleaning of every patient with topical antimicrobial agents may lead to the promotion of bacterial resistance. This is not to say that universal decolonization is not effective but that its long-term implications have not been extensively studied and may carry additional risks.

Two recommendations are emerging for the control of MRSA. The first is to screen for and treat carriers, the second is universally treat everyone and run the theoretical risk of worsening bacterial resistance and changing the microbiome of both the patient and the facility. The decade long policy followed in some, and hopefully few, facilities of not doing either of these interventions appears to no longer be an option.

## Competing interests

The authors have no conflict of interest to declare.

## Authors’ contributions

KTK conceptualized project, performed literature search, responded to reviewers and wrote first and revised drafts of manuscript; LEC contributed to the first and revised manuscript drafts, literature search, responded to reviewers; DMS contributed to first draft and revised drafts of manuscript; SKA performed literature search, contributed to first and revised drafts of manuscripts. All authors read and approved the final manuscript.
